# Evaluation of Clinical Performance of Alkasite Restorative Materials: A Systematic Review and Meta-Analysis

**DOI:** 10.3390/jfb17020093

**Published:** 2026-02-13

**Authors:** Chloé Laporte, Rim Bourgi, Carlos Enrique Cuevas-Suárez, Naji Kharouf, Louis Hardan, Miguel Ángel Fernández-Barrera, Anh Tuan Dang, Youssef Haikel, Abigailt Flores-Ledesma

**Affiliations:** 1 Department of Biomaterials and Bioengineering, INSERM UMR_S 1121, University of Strasbourg, 67000 Strasbourg, France; chloelaporte9@gmail.com (C.L.); rim.bourgi@hotmail.com (R.B.); dentistenajikharouf@gmail.com (N.K.); youssef.haikel@unistra.fr (Y.H.); 2 Department of Endodontics and Conservative Dentistry, Faculty of Dental Medicine, University of Strasbourg, 67000 Strasbourg, France; 3 Pôle de Médecine et Chirurgie Bucco-Dentaire, Hôpital Civil, Hôpitaux Universitaire de Strasbourg, 67000 Strasbourg, France; 4 Department of Restorative and Esthetic Dentistry, Faculty of Dental Medicine, Saint-Joseph University of Beirut, Beirut 1107 2180, Lebanon; louis.hardan@usj.edu.lb; 5 Dental Materials Laboratory, Academic Area of Dentistry, Autonomous University of Hidalgo State, San Agustín Tlaxiaca 42160, Mexico; miguel_fernandez10334@uaeh.edu.mx; 6 Dental Materials and Biomaterials Laboratory, Faculty of Stomatology, Meritorious Autonomous University of Puebla, Puebla 72570, Mexico; abigailt.flores@correo.buap.mx; 7 Faculty of Dentistry, Haiphong University of Medicine and Pharmacy, Haiphong 180000, Vietnam; dtanh@hpmu.edu.vn; 8 Department of Odonto-Stomatology, Haiphong Medical University Hospital, Haiphong 180000, Vietnam

**Keywords:** Cention N, dental caries, meta-analysis, restorative materials, systematic review

## Abstract

Ion-releasing restorative biomaterials have gained increasing attention in minimally invasive dentistry due to their potential to combine mechanical reliability with therapeutic functionality. Cention^®^ N is an alkasite-based restorative material designed to release fluoride, calcium, and hydroxyl ions while exhibiting mechanical properties comparable to resin-based composites. The present study aimed to systematically evaluate the clinical performance of this ion-releasing restorative material in comparison with conventional resin composites and glass ionomer cements. A comprehensive systematic search was conducted in PubMed (MEDLINE), Cochrane Library, Web of Science, Scopus, EMBASE, and SciELO databases up to 31 October 2024, following the PRISMA guidelines. Clinical studies assessing restorative performance outcomes were included. Meta-analyses were performed using Review Manager software (version 5.1). Fourteen studies met the inclusion criteria for qualitative synthesis, of which ten were eligible for quantitative analysis. The pooled results demonstrated comparable clinical performance between alkasite restoratives and resin-based composites regarding retention and secondary caries incidence, while superior outcomes were observed when compared with glass ionomer cements. Within the limitations of the available evidence, ion-releasing alkasite restorative materials represent a clinically acceptable alternative to conventional restorative options, combining functional biomaterial properties with reliable clinical performance. The conclusions should be interpreted within the context of the included studies, which exhibited clinical heterogeneity and, in several cases, a moderate risk of bias.

## 1. Introduction

Oral health is essential and an integral part of the general health of people [1]; dental caries is one of the oral diseases that little by little and over time has become one of the most recurrent pathologies of humanity [2]. Recently, approximately 88.5% of the population between 0 and 15 years old were found to have this disease [3]. The burden of oral diseases such as caries is particularly high in disadvantaged and poor population groups, both in developing and developed countries [4].

The pursuit of the ideal dental restorative material is a cornerstone of modern dentistry, balancing functional longevity with biological harmony [5]. For over a century, dental amalgam served as a ubiquitous and reliable direct restorative due to its exceptional durability, simplicity of use, and low cost [6]. However, growing concerns regarding its mercury content, environmental impact, and non-adhesive, non-esthetic nature have precipitated a global decline in its use, aligning with the Minamata Convention on Mercury [7].

This paradigm shift has accelerated the development and adoption of adhesive, tooth-colored alternatives, primarily resin-based composites (RBCs) and glass ionomer cements (GICs) [8]. RBCs, leveraging a resin matrix reinforced with inorganic fillers, have become the gold standard for esthetic restorations, offering superior mechanical properties, excellent polishability, and reliable micromechanical bonding [9,10]. Continued innovation focuses on enhancing their performance through nanotechnology and bioactive fillers, aiming to improve fracture toughness, wear resistance, and even remineralization potential [11,12,13]. Conversely, GICs are valued for their chemical adhesion to tooth structure and sustained fluoride release, which confer a caries-inhibitory effect. Nevertheless, their comparatively lower mechanical strength and fracture toughness often limit their use to low-stress-bearing situations [14,15].

Concurrently, the philosophy of minimally invasive dentistry (MID) has gained paramount importance, emphasizing maximal preservation of healthy tooth structure, early lesion detection, and therapeutic interventions [16,17]. Within this framework, the role of a restorative material extends beyond mere space filling. There is a compelling demand for “bioactive” or “smart” materials that not only restore form and function but also interact positively with the dental pulp and surrounding tissues to promote healing and remineralization and prevent recurrent disease [18].

This need has driven the development of a distinct class of materials designed to release therapeutic ions [19,20]. Among these, alkasite-based restoratives represent a notable hybrid innovation. Launched in 2016, Cention^®^ N (Ivoclar Vivadent, Schaan, Liechtenstein) is a prominent example, classified as an alkasite [21]. It is a resin-based, bulk-fill material capable of controlled release of fluoride, calcium, and hydroxyl ions, thereby creating an alkaline environment to counteract cariogenic challenges [22,23]. Mechanically, it is engineered to rival conventional RBCs, while its ion-release profile echoes the bioactive philosophy of GICs. It offers dual-cure capability and can be placed with or without a separate adhesive system, presenting clinicians with a versatile tool [24,25].

The proposed clinical benefit of Cention^®^ N stems from its dual mechanism as a restorative and therapeutic agent [26]. Its bioactive potential is primarily driven by the sustained release of fluoride (F^−^), calcium (Ca^2+^), and hydroxyl (OH^−^) ions [22]. The fluoride release is intended to inhibit bacterial metabolism and promote the remineralization of the adjacent tooth structure [27]. Concurrently, the release of calcium and hydroxyl ions increases the local pH, creating an alkaline environment that counteracts the acidic challenge from cariogenic biofilms and may further enhance remineralization [28]. This ion-release profile aims to provide a continuous, protective effect at the restoration–tooth interface, potentially reducing the risk of secondary caries—a primary cause of restoration failure [28].

Since its introduction, a growing body of clinical studies has sought to evaluate the performance of Cention^®^ N in various cavity classes (I, II, V) in both primary and permanent dentition [29,30,31,32,33]. However, the evidence remains dispersed, with studies often employing different comparators (RBCs, GICs), evaluation criteria (USPHS, FDI), and follow-up periods. A comprehensive, quantitative synthesis of this clinical data is lacking.

Therefore, this study aimed to systematically review and meta-analyze the available evidence from randomized controlled trials to evaluate the clinical performance of the alkasite restorative material Cention^®^ N, primarily in terms of retention and secondary caries incidence, compared to conventional resin composites and glass ionomer cements. The null hypothesis is that there is no significant difference in clinical performance between Cention^®^ N and these established restorative materials.

## 2. Materials and Methods

### 2.1. Data Sources

This systematic review and meta-analysis adhered to the guidelines outlined in the Preferred Reporting Items for Systematic Reviews and Meta-Analyses (PRISMA Statement) [34]. The study protocol was registered in the PROSPERO database under the identifier CRD42023354699. The following PICO framework was used: population, permanent or deciduous teeth of humans, with Class I, Class II or Class V cavities; intervention: alkasite restorative materials; control, other restorative materials; outcome, clinical performance. The research question was as follows: what is the clinical performance of alkasite-based restorative materials?

### 2.2. Search Strategy

The literature search was performed by two independent reviewers (R.B. and C.E.C.-S.) including articles up to 31 October 2024. The following databases were screened: PubMed (MEDLINE), Cochrane Wiley, Web of Science, Scopus, EMBASE and SciELO. The search strategy was developed according to the MeSH terms defined in Table 1. All studies were imported into the Rayyan QCRI mobile application [35].

### 2.3. Eligibility Criteria

The title and abstract of each identified article were reviewed by two independent reviewers (C.L. and N.K.) to determine if the article should be considered for full-text review. Manuscripts for full-text review were selected according to the following eligibility criteria: (I) clinical trials reporting the use of alkasite-based restorative materials, (II) included a control group where other restorative materials (glass ionomer, resin composite, amalgam) were evaluated, (III) included a minimum follow up of 12 months, (IV) publications in English, Spanish, or Portuguese. Exclusion criteria encompassed several publication types: case reports, case series, pilot studies, expert opinions, conference abstracts, and reviews. Two reviewers independently conducted the selection, with any discrepancies adjudicated by a third reviewer (L.H.) to reach a consensus.

### 2.4. Data Extraction

Data extraction was performed independently by two trained reviewers (A.F.-L. and M.A.F.-B.) using Microsoft Office Excel 2016 (Microsoft Corporation, Redmond, WA, USA). The reviewers recorded data from the included manuscripts in a standardized form, capturing the following variables: first author, publication year, study type, registration number, alkasite restorative material, control group, participant number and age, restoration class and substrate, evaluation criteria, and follow-up duration.

### 2.5. Quality Assessment

Two reviewers (R.B. and L.H.) independently assessed the risk of bias for the selected articles using the Cochrane RoB2 tool for randomized clinical trials [36]. They evaluated each study across five domains: (1) selection bias (random sequence generation, allocation concealment), (2) performance and detection bias (blinding of participants, personnel, and outcome assessors), (3) attrition bias (incomplete outcome data), (4) reporting bias (selective reporting), and (5) other biases (including protocol registration in CONSORT). Each domain was then classified as having a low, unclear, or high risk of bias.

### 2.6. Statistical Analysis

All meta-analyses were conducted in Review Manager Software (RevMan, The Cochrane Collaboration, Copenhagen, Denmark), version 5.1. A fixed-effects model was employed for the global analysis to generate pooled-effect estimates. The primary comparison was the risk difference in restoration retention and secondary caries between alkasite materials and other restorative materials. For this analysis, outcomes from each study were dichotomized as either “acceptable” (Alpha or Bravo scores) or “unacceptable” (Charlie or Delta scores in any assessed characteristic). The risk difference was then calculated using the prevalence of unacceptable restorations (events) and the total number of restorations per group. A fixed-effects model was initially chosen for the global analysis due to the assumption of a common effect size.

In order to quantify the effects of different outcomes, we performed separate random-effect meta-analyses when the alkasite restorative material was used with or without adhesive, and subgroups were assessed according to the restorative material used as comparator: resin composite or glass ionomer cement. Data were summarized into the following follow-ups: 6–12 months and >12 months. In case a study reported data twice within the range described above, data from the longest follow-up period were used. A *p* value ≤ 0.05 was considered statistically significant. Statistical heterogeneity among studies was assessed using the I^2^ statistic. An I^2^ value > 50% was considered to represent substantial heterogeneity.

## 3. Results

**Figure 1:**
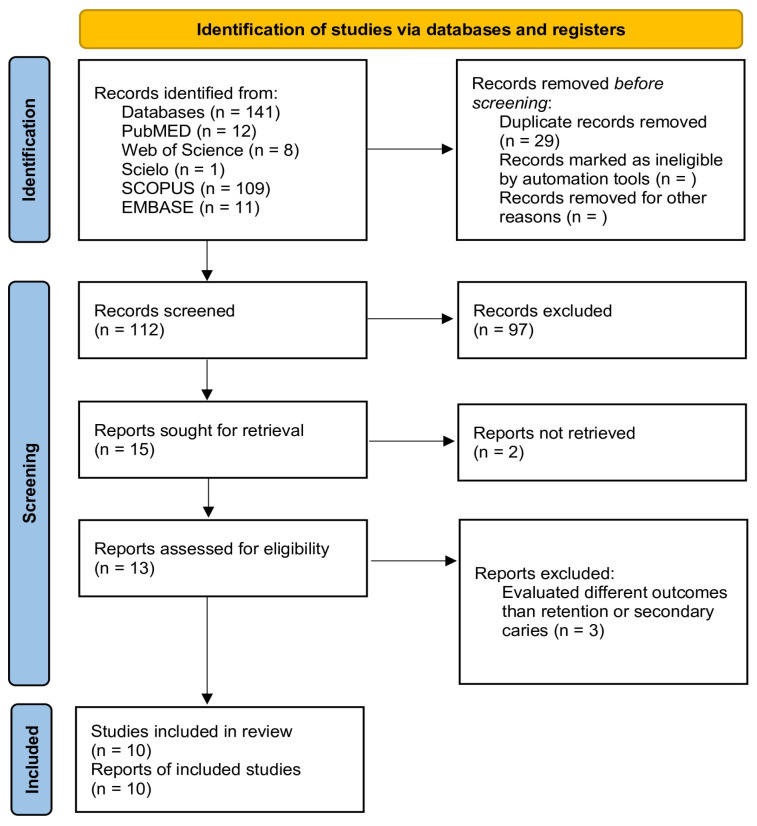
PRISMA flowchart.

A total of 141 publications were retrieved from all databases. After duplicates were removed, 112 manuscripts were assessed for the initial examination. Of these, 97 studies were excluded after reviewing the titles and abstracts. In total, 15 studies were evaluated by full-text reading. Of these, two studies were excluded since a full-text file could not be obtained [37,38]. A total of 13 studies were included in the qualitative analysis [29,30,31,32,33,39,40,41,42,43,44,45,46]. From these, 10 studies were included in the meta-analysis. Figure 1 shows the PRISMA flow diagram for the selection of the studies.

The qualitative analysis of the studies incorporated in this systematic review is summarized in Table 2. The articles collected for this study were from 2018 to 2024; from the present studies, some of the registration numbers were obtained, and we analyzed the clinical performance of Cention^®^ N in class I and II restorations, including both primary and permanent dentition against other restorative materials, such as glass ionomer and resins. Among the evaluation systems used to assess restorations were the World Dental Federation (FDI) criteria, the modified Cvar and Ryge criteria, the modified United States Public Health Service (USPHS) criteria, and the original United States Public Health Service (USPHS) criteria. The maximum follow-up duration recorded among the included studies was 17 months, and the main clinical outcomes of each study were reported accordingly.

**Figure 2:**
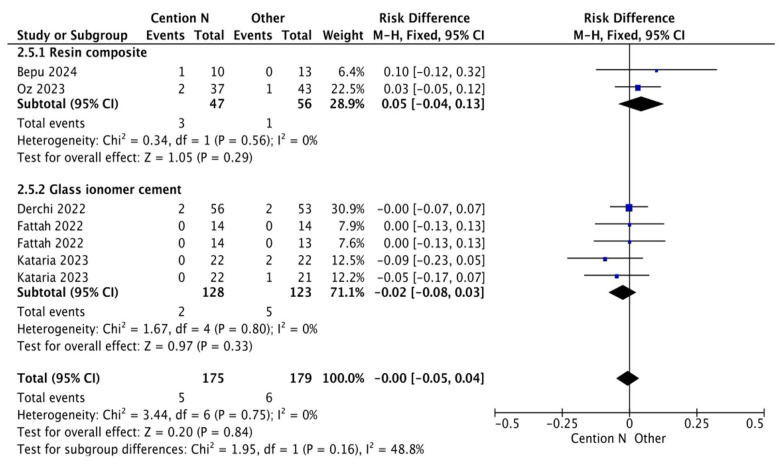
Results from the meta-analysis of the retention at <12 months of follow-up when Cention^®^ N was used without adhesive [31,32,33,42,44].

**Figure 3:**
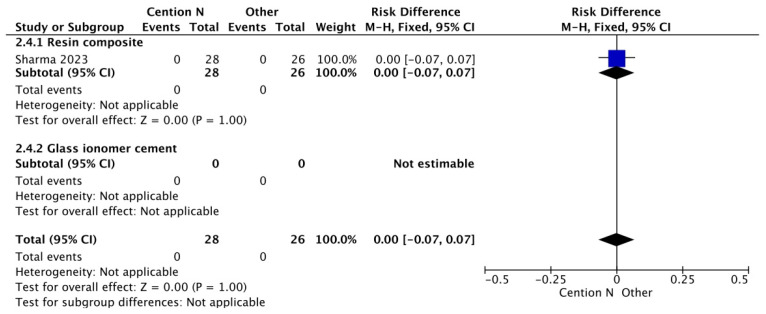
Results of the meta-analysis of the retention at >12 months of the Cention^®^ N with adhesive compared to other restorative materials [40].

**Figure 4:**
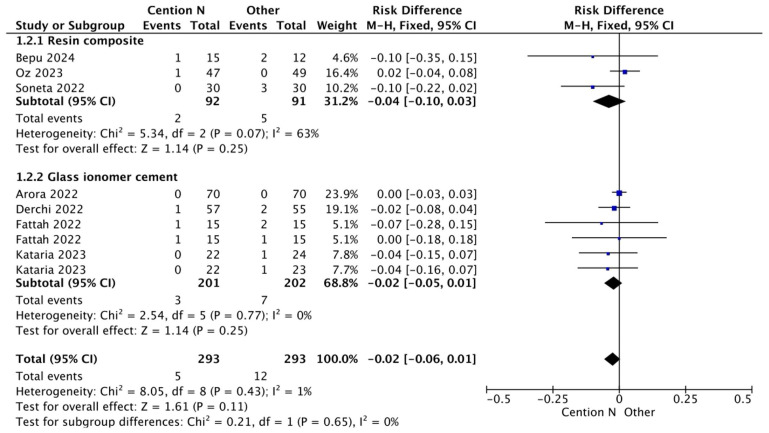
Results from the meta-analysis of the retention rate at >12 months of follow up of Cention^®^ N without an adhesive system [29,31,32,33,42,44,45].

**Figure 5:**
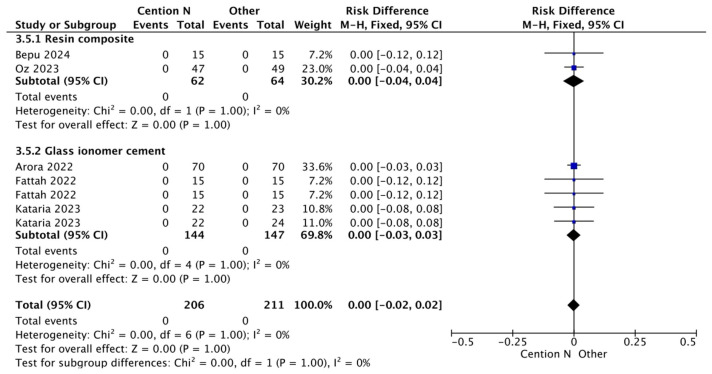
Results from the meta-analysis for the assessment of secondary caries at >12 months for Cention^®^ N used with an adhesive system [29,31,32,33,42].

**Figure 6:**
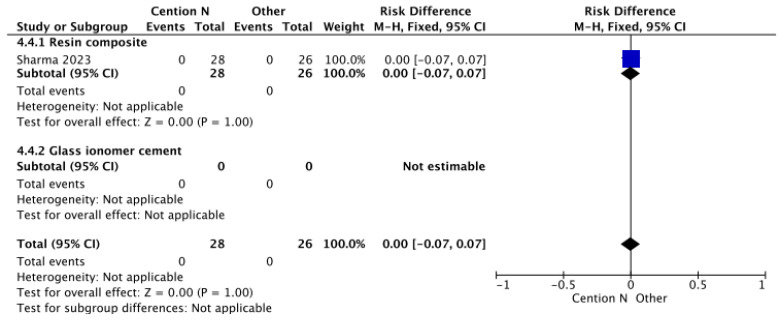
Results from the meta-analysis for the assessment of secondary caries at >12 months for Cention^®^ N used without an adhesive system [40].

**Figure 7:**
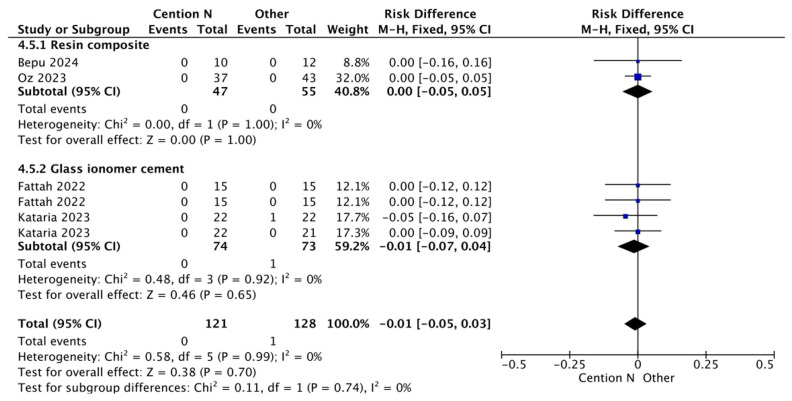
Results from the meta-analysis for the assessment of secondary caries at <12 months for Cention^®^ N used with an adhesive system [31,32,33,42].

The results for the meta-analysis are presented in Figure 2, Figure 3, Figure 4, Figure 5, Figure 6 and Figure 7. It is important to note that non-significant differences (*p* > 0.05) in the following analyses do not definitively prove equivalence. These findings may also reflect limited statistical power, clinical heterogeneity, or variability inherent in the included studies. Figure 2 shows the results for the analysis of the retention at <12 months of follow-up when the alkasite restorative material was used without adhesive; the global analysis showed that there were no statistically significant differences between Cention^®^ N and other restorative materials (*p* = 0.11).

The risk-of-bias assessment across the included studies reveals varying levels of methodological rigor. Several studies, including those by Bepu [31], Oz [32], Kataria [33], Arora [29], Mushtaq [46], and Hirani [30], demonstrated a high risk of bias, particularly in adherence to intervention and missing outcome data, raising concerns about their reliability. Studies such as those by Mustafa [39], Sharma [40], Da Cunha [43], Derchi [44], and Soneta [45] exhibited some concerns, mainly related to randomization, adherence to intervention, and selection of reported results, indicating moderate risk. On the other hand, Kaur [41] and Fattah [42] were categorized as low-risk studies, with minimal methodological concerns. Overall, while some studies maintain robust methodological integrity, others exhibit significant bias, necessitating cautious interpretation of their findings (Table 3).

## 4. Discussion

In this study, a systematic review with meta-analysis was conducted to analyze the clinical performance of alkasite-based restorative materials compared to other restorative materials. According to the results, none of the comparisons showed statistically significant differences between Cention^®^ N and composite resin or glass ionomer.

According to the results, the retention rate of Cention^®^ N beyond 12 months of follow-up, when placed without adhesive, is similar to the retention rate of both composite resins and glass ionomer. Cention^®^ N, considering anatomical form and marginal adaptation criteria, demonstrates good retention, anatomical form, marginal adaptation, control of cariogenic activity, and reduced discoloration [47], resulting in strong mechanical properties and good long-term stability [29], which could explain the clinical behavior of this material.

A similar trend is observed in the follow-up results beyond 12 months (Figure 3). This behavior of the materials (Cention^®^ N, glass ionomer, and resins) can be explained by the adhesive system used, which includes the 10-methacryloyloxydecyl dihydrogen phosphate monomer. A key property of this monomer is its ability to protect collagen fibers from degradation through the formation of stable calcium salts. Furthermore, it modulates the polymer matrix by balancing hydrophilic and hydrophobic domains, thereby optimizing substrate wetting and enabling effective copolymerization [48], thus influencing the performance of these materials.

In the case of Figure 4, the comparison of retention rates among restorative materials shows similarities, particularly between light-cured composite resin and Cention^®^ N without adhesive over a period longer than 12 months. The mechanical properties of Cention^®^ N have been attributed to the presence of filler particles composed of barium–aluminum silicate glass and calcium–aluminum silicate, as well as the crosslinked polymer structure [40]. On the other hand, conventional resin composites are characterized by a high filler load and reduced particle size, which decrease interstitial spacing. This microstructure helps protect the resin matrix and reduces filler particle dislodgement, thereby improving wear resistance and surface texture retention [49,50]. The alkasite material and this nanocomposite both demonstrate wear resistance comparable to nanohybrid and bulk-fill composites [50], a property that contributes to their clinically acceptable anatomical form in restorations.

The comparable clinical performance, particularly in retention and marginal integrity, can be attributed to fundamental material properties. While Cention^®^ N relies on its alkaline filler and stress-relieving Isofillers [51], modern RBCs achieve performance through high filler load (~70–80 wt.%), optimized monomer chemistry (e.g., low-shrinkage monomers like Bis-EMA, UDMA), and sophisticated filler–matrix coupling [52]. These features collectively reduce polymerization shrinkage stress, enhance fracture toughness, and improve wear resistance—key determinants of long-term clinical success [53]. Therefore, the similar clinical outcomes observed suggest that alkasite materials have successfully engineered a property profile that meets the clinical benchmarks set by advanced RBCs.

Figure 5 presents a comparative analysis of secondary caries within a period of less than 12 months between the use of light-cured composite as a restorative material and Cention^®^ N without adhesive. Cention^®^ N forms a dense, highly crosslinked polymer network that achieves complete polymerization through the entire restoration. This material incorporates a patented filler, which functions as a stress-relieving component. By absorbing internal stresses, the Isofiller mitigates the forces that cause low-volume shrinkage, thereby significantly reducing the potential for microleakage [54]. A study by Mazumdar et al. (2019) emphasized that Cention^®^ N exhibited minimal microleakage compared to GIC and amalgam in an in vitro setting [54], resulting in no statistically significant differences between the materials.

In Figure 6, the analysis of secondary caries within 12 months using a glass ionomer-based restorative material compared to Cention^®^ N with adhesive suggests that the presence of Isofillers—components that function as anticorrosive primers and adhesion promoters—acts as a shrinkage stress reducer, keeping contraction forces to a minimum and thus preserving dental integrity [55]. Consequently, no statistically significant differences were observed.

Figure 7 shows a result like the previous figures regarding the analysis of secondary caries when comparing a glass ionomer-based restorative material and Cention^®^ N without adhesive over a period of less than 12 months. This outcome can be attributed to GIC, which is well known for its fluoride release, with a standard release of 5.11 parts per million (ppm) after seven days post-operation [56]. Cention^®^ N also releases a substantial amount of fluoride, reaching 7.94 ppm in an acidic medium after seven days [36], which is higher than traditional GIC [57]. Statistical analysis revealed no significant differences between the two materials.

This outcome can be attributed to the fluoride-releasing capacity of both materials. GIC is well-known for its sustained fluoride release via an acid–base reaction [58]. Cention^®^ N employs a different mechanism, where fluoride is incorporated into its filler system (e.g., ytterbium fluoride) and released through a diffusion-controlled process, often demonstrating a higher initial burst release in acidic environments [28]. This comparable bioactive functionality, albeit through different release mechanisms, may underlie the similar clinical outcomes observed for secondary caries prevention.

From a clinical perspective, the finding of comparable performance between alkasite restoratives and resin composites suggests that Cention^®^ N can be considered a viable alternative for Class I and II restorations, particularly where its additional ion-releasing property is deemed beneficial. Its performance appears more consistent than that of conventional GICs in these stress-bearing situations. The option to use it without a separate adhesive may offer a simplified, time-efficient procedure in select cases, though the long-term implications of this technique require further study.

It is important to differentiate the alkasite material evaluated here (Cention^®^ N) from the emerging class of ‘self-healing’ bioactive materials. While both aim to extend restoration longevity, their mechanisms differ fundamentally. Alkasites like Cention^®^ N are primarily ion-releasing materials, providing a continuous therapeutic release of fluoride, calcium, and hydroxide ions to inhibit demineralization and promote a remineralizing environment. In contrast, ‘self-healing’ composites typically incorporate microcapsules or vascular networks that release healing agents (e.g., new monomers) upon crack formation, aiming to autonomously repair micro-damage and restore mechanical integrity. The former focuses on chemical biofilm management and tooth preservation, while the latter focuses on physical restoration durability. Both represent significant advances, but they target different failure mechanisms. Future materials may seek to combine these approaches.

## 5. Conclusions

Within the limitations of the available evidence, which includes studies with moderate risk of bias and follow-up periods generally under two years, this meta-analysis suggests that the alkasite restorative material Cention^®^ N performs comparably to resin-based composites in terms of retention and secondary caries incidence for Class I and II restorations and may offer a clinical advantage over conventional glass ionomer cements. Therefore, based on current short-to-medium-term data, Cention^®^ N represents a clinically acceptable bioactive alternative. Future high-quality, long-term randomized trials are needed to confirm these findings and establish its performance over extended service periods.

## Figures and Tables

**Table 1:** Keywords used for the literature search in PubMed.

Number	Terms Used
1	Alkasite OR Cention-N OR alkasite restorative OR Cention N OR Alkasite material OR Cention-N OR Alkasite filling OR Bulk-fill alkasites cement OR Alkasite restoration OR Alkasite cement OR Alkasite restorative material
2	Controlled Clinical Trial OR Retrospective Studies OR Randomized Controlled Trial OR Retrospective Study OR Prospective Studies OR Prospective Study OR Clinical Trial OR Randomized clinical trial
1 and 2	Alkasite OR Cention-N OR alkasite restorative OR Cention N OR Alkasite material OR Cention-N OR Alkasite filling OR Bulk-fill alkasites cement OR Alkasite restoration OR Alkasite cement OR Alkasite restorative material AND Controlled Clinical Trial OR Retrospective Studies OR Randomized Controlled Trial OR Retrospective Study OR Prospective Studies OR Prospective Study OR Clinical Trial OR Randomized clinical trial

**Table 2:** Main characteristics of the studies included in the qualitative synthesis.

First Author	Type of Study and Registration Number	Alkasite Restorative Material	Control Group	Number of Participants and Age	Class and Substrate	Restoration Evaluation Criteria Used	Follow-Up	Main Results
Bepu 2024 [31]	Randomized clinical trial—no registration number	Cention^®^ N (Ivoclar Vivadent).	Tetric N (Ceram Bulk Fill, Ivoclar Vivadent)	33 (with a mean age of 38)	Class I and Class II (permanent molars)	USPHS	7 days, 6 months, and 17 months	It is feasible to restore dental teeth with root canal treatment using alkasites.
Mustafa 2024 [39]	Randomized clinical trial—no registration number	Cention^®^ N	RM-GIC	9 (18 o + years)	Periodontal conditions adjacent to restorations	The BOP, PI and PD indices	1 week, 3, 6 and 9 months	The performances of the nanofilled composite and alkasite were clinically acceptable and comparable.
Oz 2023 [32]	Randomized Clinical trial Registered at ClinicalTrials.gov (NTC04825379)	Cention^®^ N	G-ænial Posterior	31 (30 years)	Class II, (permanent molars)	USPHS	1 week, 6 and 12 months	Clinical performances of the alkasite restorative and the resin composite were similar and both the materials showed good survival.
Kataria 2023 [33]	Randomized clinical trial—no registration number	Cention^®^ N	Ketac Universal	43 (3–8 years)	Primary molars	USPHS	1 week, 6 and 12 months	The three materials that were compared are clinically acceptable and comparable in this study.
Sharma 2023 [40]	Randomized clinical trial. Clinical Trial registry of India, Reg. No.: CTRI/2020/12/029830	Cention^®^ N (light-cured) with Tetric N-Bond Universal (Selective etching)	Filtek™ Z350XT/Single bond 2 (Total-etch)	38 (7–13 years)	Class I, (permanent molars)	USPHS	12 months	The performances of the nanofilled composite and alkasite were clinically acceptable and comparable.
Kaur 2023 [41]	Randomized clinical trial—clinical trial registry in India CTRI/2023/01/048831	Cention^®^ N	Stainless steel crowns (3M ESPE, SSC)	60 (4–8 years)	Pulpotomized primary molars	USPHS	6, 9, 12 months	The performance of stainless-steel crowns (SSCs) and Centon-N materials are clinically acceptable and there is no significant difference between the two.
Fattah 2022 [42]	Randomized Clinical trial No registration number	Cention^®^ N	Nano-ionomer (Ketac Nano) Bioactive glass filler (Activa bioactive)	15 (18–50 years)	Class I (permanent teeth)	FDI	3, 6, 12 months	The three restorative materials present a good clinical performance against class I lesions with similar successes. Glass ionomer presented slightly superior biological properties compared to the other restorations.
Arora 2022 [29]	Randomized clinical trial—No registration number	Cention^®^ N (Ivoclar Vivadent, Leuven)	Posterior high-strength glass ionomer cement GC (Gold label H.S)	70 (5–8 years)	Class II (primary molars)	USPHS	3, 6, 9 months	The restorations were carried out satisfactorily over more than 9 months. Therefore, they can be considered as a clinically appropriate option for treating cavities Class 1.
Da Cunha 2022 [43]	Randomized clinical trial—REBEC number U1111-1198-3193	Cention^®^ N (Ivoclar Vivadent, Schaan, Lichenstein) with Tetric N-bond Universal (Self-etch mode)	Vitremer (3M Oral Care, St. Paul, MN, USA)	27 (4–9 years)	Class I and Class II (primary molars)	ART restoration criteria	3, 6, 12 months	All the materials evaluated are suitable for restoring occlusal cavities, but it is necessary to use adhesive in the cavities, otherwise the market will see the performance of the Census N without adhesive after 12 months of follow-up.
Derchi 2022 [44]	Randomized clinical trial—no registration number	Cention^®^ N, Ivoclar Vivadent AG, Liechtenstein	Fuji-IX, GC Europe	45 (5–9 years)	Class II (primary molars)	FDI	3, 6, 12 months	Similar performance and efficiency were shown between both materials in the restoration of residual teeth, proving to be a good alternative to the glass ionomer.
Soneta 2022 [45]	Non-randomized clinical trial—no registration number	Cention^®^ N	Posterior high-strength glass ionomer cement (GC Gold label, Fuji)	60 (6–12 years)	Class 1 (permanent molars)	VAS	1, 3, and 6 months	Both materials showed good retention and antibacterial properties, but the Cention^®^ N showed the best result.
Mushtaq 2021 [46]	Randomized clinical trial—no registration number	Cention^®^ N (Ivoclar)	Glass ionomer cement (GC Fuji IX). Composite restoration bonded with etch and rinse adhesive (Tetric-N Ceram/Tetric-N bond). Composite restoration bonded with self-etch adhesive (Tetric-N Ceram/Tetric-N bond SE)	160 (above 18 years)	Class I (permanent molars)	VAS	24, 48 h, 7 days	The performance resin composite, glass ionomer and Cention^®^ N site were clinically acceptable and comparable.
Hirani 2018 [30]	Randomized clinical trial—no registration number	Cention^®^ N	Equia Forte (GC America INC)/G-COAT PLUS, GC) Activa, Bioactive restorative	144 (18–45 years)	Class I (permanent teeth)	Postoperative sensitivity	24 h, 1 week, 1 month	The performance of Activia, Equia Forte and Cention^®^ N present similar results.

**Table 3:** Risk of bias assessment.

Studies	Year	Randomization	Assignment to Intervention	Adhering to Intervention	Missing Outcome Data	Measurement of the Outcome	Selection of the Reported Result
Bepu 2024 [31]	2024	Low risk	Some concerns	High risk	Low risk	Low risk	Low risk
Mustafa 2024 [39]	2024	Some concerns	Low risk	Some concerns	Low risk	Some concerns	Some concerns
Oz 2023 [32]	2023	Low risk	Some concerns	High risk	Some concerns	Low risk	Low risk
Kataria 2023 [33]	2023	Low risk	Low risk	High risk	Low risk	Low risk	Low risk
Sharma 2023 [40]	2023	Some concerns	Some concerns	Low risk	Low risk	Low risk	Low risk
Kaur 2023 [41]	2023	Low risk	Low risk	Low risk	Low risk	Low risk	Some concerns
Fattah 2022 [42]	2023	Low risk	Low risk	Some concerns	Low risk	Low risk	Low risk
Arora 2022 [29]	2022	Low risk	Low risk	High risk	Low risk	Low risk	Low risk
Da Cunha 2022 [43]	2022	Low risk	Low risk	Low risk	Some concerns	Some concerns	Some concerns
Derchi 2022 [44]	2022	Some concerns	Some concerns	Low risk	Low risk	Some concerns	Some concerns
Soneta 2022 [45]	2022	Low risk	Low risk	Low risk	Low risk	Some concerns	Some concerns
Mushtaq 2021 [46]	2022	Some concerns	Low risk	Some concerns	Low risk	Low risk	Low risk
Bepu 2024 [31]	2021	Low risk	Low risk	High risk	Low risk	Some concerns	Low risk
Hirani [30]	2018	Some concerns	High risk	High risk	Low risk	Some concerns	Low risk

## Data Availability

The raw data supporting the conclusions of this article will be made available by the authors on request.
